# Public sector physiotherapists’ organisation and profile: Implications for intensive care service

**DOI:** 10.4102/sajp.v79i1.1803

**Published:** 2023-03-27

**Authors:** Farhana Karachi, Rik Gosselink, Susan Hanekom

**Affiliations:** 1 Department of Physiotherapy, Faculty of Community and Health Science, University of the Western Cape, Cape Town, South Africa; 2 Division of Physiotherapy, Department of Health and Rehabilitation Sciences, Stellenbosch University, Cape Town, South Africa; 3 Department of Rehabilitation Sciences, Faculty of Respiratory Rehabilitation, KU Leuven, Leuven, Belgium

**Keywords:** physiotherapy profile, organisation, intensive care, service-delivery, public hospitals, South Africa

## Abstract

**Background:**

Physiotherapists are essential in the management of hospitalised patients. The way in which a physiotherapy service is offered in intensive care units (ICUs) can affect ICU patient outcomes.

**Objectives:**

To provide a clear picture of the organisation and structure of physiotherapy departments, the number and types of ICUs requiring physiotherapy services and the profile of physiotherapists working in South African public-sector central, regional and tertiary hospitals that house Level I–IV ICUs.

**Method:**

Cross-sectional survey design using SurveyMonkey, analysed descriptively.

**Results:**

One hundred and seventy units (the majority Level I, functioning as mixed [37%, *n* = 58] and neonatal [22%, *n* = 37] units) are serviced by 66 physiotherapy departments. The majority of physiotherapists (61.5%, *n* = 265) were younger than 30 years, had a bachelor’s degree (95.1%, *n* = 408) and were employed in production Level I and community service posts (51%, *n* = 217) with a physiotherapy-to-hospital-bed ratio of 1:69.

**Conclusion:**

Insight into the organisational structure of physiotherapy departments and physiotherapists working in public-sector hospitals with ICU facilities in South Africa was provided. It is evident that physiotherapists employed within this sector are young and early in their career development. The large number of ICUs functioning within these hospitals and high bed-to-physiotherapist ratio is concerning, highlighting the high burden of care within this sector and the possible effect on physiotherapy services in the ICUs.

**Clinical implications:**

A high burden of care is placed on public-sector hospital-based physiotherapists. The number of senior-level posts within this sector raises concern. It is not clear how the current staffing levels, physiotherapist profile and structure of hospital-based physiotherapy departments affect patient outcomes.

## Introduction

South African healthcare resources are limited. It is estimated that the public health sector receives 13.5% of the country’s total budget (Naidoo, Singh & Lalloo 2013). The public-sector healthcare system functions at the community, district, provincial and national levels, with forecasted data from Matsoso & Strachan (2011) suggesting that the ratio of healthcare worker: population would be 1:1000 in SA compared to 10:1000 in Europe in 2017 implying increased financial and human resources required. The limited healthcare resources have been redistributed from tertiary-level care to primary healthcare, with the aim of meeting the increased need for health promotion and prevention (Coovadia et al. 2009; Fusheini & Eyles 2016; Morris et al. 2021). Level I, II and IV (high care or stand down) intensive care units (ICU) facilities are situated in central, regional and tertiary public hospitals (Republic of South Africa 2011). Level I units are located in public-sector hospitals, which are affiliated to universities and have sophisticated equipment able to manage a wide spectrum of critical illnesses. These ICUs are closed, and round-the-clock care is directly managed by intensivists in collaboration with other support staff and services. These units have a medical director, 24-h medical specialists, residents and medical officers, with a nurse–patient ratio of 1:1 or 1:2. In addition to a restructured healthcare system, the public healthcare sector in SA is overburdened because of the heavy burden of disease and the large part (82%) of the population being dependent on public healthcare in the country (Obuaku-Igwe 2016).

The coronavirus disease 2019 (COVID-19) pandemic challenged existing critical care resources; highlighted ethical dilemmas around access; focused attention on staffing issues; demonstrated the potential value of telemedicine; and brought to the fore the continued plight of ICU survivors and their families (Amass et al. 2020; Cleary et al. 2021; Sevin et al. 2018). Within the critical care community, warnings had emerged, even before the pandemic, that a multifaceted approach to strengthen early critical care services in low-resource settings is required (Cichowitz et al. 2018; Hlafa, Sibanda & Hompashe 2019; Losonczy et al. 2021; Marshall et al. 2017). Evidence also emerged from a South African unit that the way in which a physiotherapy service is provided in ICU can improve patient outcomes (Hanekom, Louw & Coetzee 2012).

Physiotherapists form part of the multidisciplinary healthcare team and play an essential role in the management of hospitalised patients (Ntinga & Van Aswegen 2020; Scribante, Schmollgruber & Nel 2004). The profession is integral to health promotion and prevention, acute care (intensive or critical care) and rehabilitation. Physiotherapists are trained to provide quality care which could improve patient outcomes with minimal cost to the healthcare budget, thereby improving efficiency in the healthcare system (Morris et al. 2021). However, globally, many variations in the role and practices of physiotherapists in the management of patients in intensive care have been reported (Berney, Haines & Denehy 2012; Lottering & Van Aswegen 2016; Norrenberg & Vincent 2000; Shpata, Kreka & Tani 2021). While work has been done internationally and locally to define and standardise the physiotherapy provided in ICU (Hanekom et al. 2015; Skinner et al. 2016; Twose, Jones & Cornell 2019; Van Aswegen et al. 2017), it is important to understand the context within which hospital-based physiotherapy departments, who are responsible for providing a service to ICUs in SA, function.

Staff shortages and limited numbers of skilled healthcare professionals may affect quality of care and patient outcomes (Garland & Gershengorn 2013; Mackay & Ellis 2002; Hinshaw 2011; Ntinga & Van Aswegen 2020). There is a paucity of published reports or papers on the age range; job ranks; qualifications; involvement in student supervision and training; and continuing professional development of public-sector physiotherapists in SA. The aim of this paper is to provide a clear picture of the organisation and structure of physiotherapy departments, the number and types of ICUs that need physiotherapy service provision and the profile of the physiotherapists working in the public-sector hospitals with existing ICU facilities in SA.

## Method

An exploratory descriptive cross-sectional survey design included South African public-sector central, regional and tertiary hospitals that house Level I–IV ICUs, and the physiotherapy departments situated in these hospitals providing ICU services.

### Study population and sampling strategy

All physiotherapy departments situated in the central, regional and tertiary public hospitals housing ICU facilities in SA were eligible for inclusion, but district and specialised hospitals were excluded. The Government Gazette of South Africa (Republic of South Africa 2011) was used to identify the central, regional and tertiary hospitals in each of the nine provinces in SA, and those specifically with ICUs were identified through direct communication with the hospital administrator or CEO and were included in our study. A total of 66 hospitals with ICUs and physiotherapy departments were identified. A total population sampling method was used, including all 66 hospitals and their physiotherapy departments. The heads of the 66 physiotherapy departments were included as participants in our study.

### Data collection

An electronic self-reporting survey (online supplement) was designed on SurveyMonkey (Momentive, Inc., San Mateo, California, United States) by the first author, F.K. The survey included questions used in previous international surveys and was designed to address the specific objectives of our study (Berney et al. 2013; Norrenberg & Vincent 2000; Shpata et al. 2021; Skinner et al. 2008). Questions on the organisation and structure included questions on the number of hospital beds in the hospital in which the department was based; who is responsible for running the physiotherapy department; the total number of physiotherapists working in the respective departments; the number of physiotherapists in each job rank category (e.g. production Level I); permanent or contract employees; the involvement of the departments in training and supervision of student physiotherapists in intensive care; and the involvement of the departments in intensive care–related continual professional development (CPD) activities. A question regarding the types of ICUs situated in the hospitals in which the respective physiotherapy departments were based was also included in the survey to determine the number and type of ICUs to which the physiotherapists render services. Questions on the profile of the physiotherapists included questions on the age categories of the physiotherapists in the departments; qualifications; training; ICU clinical education block training as student physiotherapists; and international intensive care work experience.

### Face and content validity

The survey and objectives were sent via e-mail through SurveyMonkey to a group of four national and international academic and clinical experts in ICU physiotherapy to ensure face and content validity. They were asked to review the survey questionnaire and state whether the questions were appropriate to meet the objectives of our study. Adaptations included adding additional questions and adjusting some questions to meet all our study objectives. The final version of the survey was piloted for ease of use and time to complete. Three clinical physiotherapists who previously had worked in the public sector were included in this pilot. The survey took between 5 min and 10 min to complete online via SurveyMonkey and 10 min to 15 min via telephone. Each hospital was contacted telephonically by the first author to determine the existence of ICUs and a physiotherapy department, and the contact details (telephone number and e-mail address) of the head of department (HOD) of each physiotherapy department were obtained. Verbal consent was obtained from the HODs. Survey links embedded in an e-mail were sent via the SurveyMonkey platform. Respondents were asked to complete this survey within 5 days. To increase the response rate, two 1-week reminders were given, after which a telephonic interview to complete the survey was scheduled.

### Data capturing and analysis

All survey data submitted by participants was automatically entered and stored in a Microsoft Excel database (Microsoft Corporation, Redmond, Washington, United States) on the Survey Monkey platform. Data were coded in the Excel data sheet by the research assistant, and the coded data were checked and verified by the first author. All surveys returned formed part of the analysis. All returned surveys were fully completed. Data were analysed by the first author in consultation with a statistician using the Statistical Package for Social Sciences (SPSS), version 24 (IBM Corporation, Armonk, New York, United States). The response and completion rates were calculated in percentages. Descriptive data analysis was carried out. Categorical and continuous data were summarised as frequencies and percentages and means (SD) or medians (IQR), respectively, and were presented in text, tables and figures, as indicated. Inferential statistics were calculated, using nonparametric one-sample chi-square tests for categorical variables; the independent-samples Kruskal–Wallis test for categorical and continuous data comparison; and the one-sample binomial test for dichotomous variables to test for the probability of equal responses. Results were significant at a *p*-value of 0.05, two-sided.

### Ethical considerations

The project was registered with the institutional review board (ref. no. S13/09/170) of Stellenbosch University. Permission was obtained from the Departments of Health of each of the nine provinces, together with permission from the hospital chief executive officers, Research Ethics Committees or heads of Physiotherapy Departments of the included hospitals, as required. All responses were captured on the password-protected SurveyMonkey platform. Anonymity and confidentiality were protected as the data were only accessible to the first author.

## Results

**FIGURE 1:**
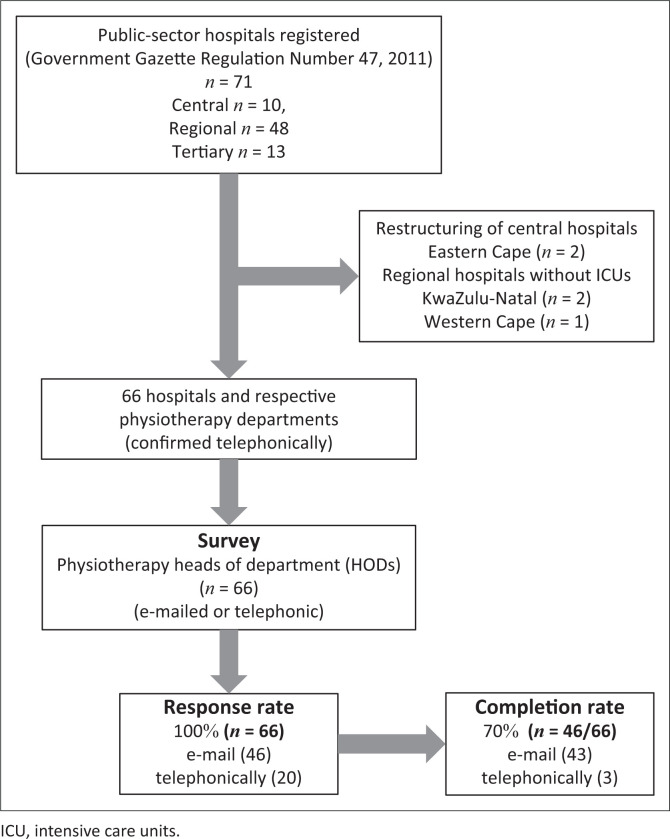
Flow diagram of participants.

A total of 10 central, 48 regional and 13 tertiary public-sector hospitals were identified from the Gazette (Figure 1). All nine provinces took part in the survey, with all provincial Departments of Health providing permission to contact the included hospitals and their relevant physiotherapy departments. In the Eastern Cape province, four central hospitals had joined to form two central hospital complexes, and thus this province had a total of four instead of six hospitals. Three regional hospitals, one in the Western Cape and two in KwaZulu-Natal, reported having no ICUs. A population sample of 66 hospitals was obtained and included. All heads of the physiotherapy departments (*n* = 66, 100%) responded to the survey, as per Figure 1.

A total of 170 public-sector ICUs were reported by the heads of department, with a physiotherapy department in each of the 66 included hospitals. Table 1 shows the geographical distribution and different types of ICUs reported by 100% (*n* = 66) of the physiotherapy departments in each province. A large number of ICUs were found in Gauteng and KwaZulu-Natal provinces, which also have the most hospitals. The majority of the units are Level I units functioning as mixed (37%, *n* = 58) and neonatal units (22%, *n* = 37). All provinces have access to Level I units.

**TABLE 1:** Distribution of intensive care units and hospitals across the nine provinces.

Province	Medical	Medicosurgical (mixed)	Paediatric	Respiratory	Neonatal	Burns	Cardio thoracic	Neuro surgical	Surgical	Trauma	Other†	Total ICUs/province	Total number of hospitals
Eastern Cape	0	6	3	0	5	0	1	0	0	0	0	15	4
Free State	1	6	2	0	5	0	1	1	1	0	0	17	6
Gauteng	2	13	3	1	9	2	3	3	1	3	1	41	15
KwaZulu-Natal	3	12	5	1	9	2	1	1	3	1	4	42	16
Limpopo	0	6	2	0	2	2	0	0	0	0	1	13	7
Mpumalanga	0	4	1	0	1	0	0	0	0	0	0	6	5
Northern Cape	0	5	1	0	3	0	0	0	0	0	0	9	2
North West	0	2	1	0	1	0	0	1	0	0	1	6	5
Western Cape	2	4	1	1	2	1	2	2	2	2	2	21	6

**Total ICUs**	**8**	**58**	**19**	**3**	**37**	**7**	**8**	**8**	**7**	**6**	**9**	**170**	**66**

ICU, intensive care units.

† , Coronary, Obstetrics and Gynaecology, Acute Spinal Cord or Renal.

**FIGURE 2:**
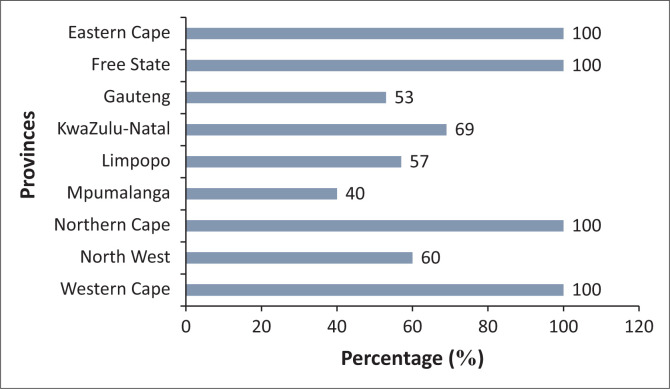
Survey completion rate per province (*N* = 66).

While 100% of the physiotherapy department heads had responded to the survey and answered the questions related to types of ICU via e-mail (70%, *n* = 46) or telephonically (30%, *n* = 20) (Figure 1), 70% (*n* = 46) of the physiotherapy department heads completed the full survey. The completion rate per province is shown in Figure 2. Although Gauteng, KwaZulu-Natal, the Western Cape and North West provinces had the highest percentages of total respondents who completed the survey, the responses from the provinces occurred with equal probability (*p* = 0.09). Mpumalanga had the lowest response rate (Figure 2).

### Departmental functioning

All physiotherapy departments participating in the survey (100%, *n* = 46) were run and organised by a qualified and registered physiotherapist. Of the physiotherapy departments, 59% (*n* = 27) were based in hospitals that had between 400 and 1000 beds; 28% (*n* = 13) in hospitals with < 400 beds; 9% (*n* = 4) in hospitals with between 1000 and 1500 beds; and 4% (*n* = 2) in hospitals with ≥ 1500 beds.

All departments provided physiotherapy services in the hospital ICUs. Student training and supervision in intensive care was reported to be provided by more than half (52%, *n* = 24, *p* = 0.883) of the responding physiotherapy departments. The majority of physiotherapy departments (74%, *n* = 34, *p* = 0.002) reported that all the physiotherapists working in their departments were trained in South Africa, and the majority had completed a clinical rotation in intensive care as student physiotherapists (93%, *n* = 43, *p* < 0.001).

### The physiotherapists

A total of 429 physiotherapists worked in the responding physiotherapy departments, with a total of 50 physiotherapy assistants. The total number of beds in the hospitals from which responses were obtained is 29 663 (Republic of South Africa 2011). The physiotherapist-to-bed ratio in the public-sector hospitals completing the survey was thus calculated as one physiotherapist for every 69 beds.

The majority of physiotherapists (61.5%, *n* = 265) working in the surveyed hospitals were younger than 30 years of age. The most common qualification (95.1%, *n* = 408) was a bachelor’s degree. Only one (0.2%) physiotherapist was reported to have a masters’ degree, specifically in the area of critical care. Half of all the physiotherapy staff working in public-sector hospitals in South Africa were appointed either as production Level 1 junior (31%, *n* = 133) or community service therapists (20%, *n* = 84). Of the 429 therapists working in the public-sector hospitals surveyed, 23% worked in KwaZulu-Natal (*n* = 99) and 25% in Gauteng (*n* = 107), and 8% (*n* = 34) of physiotherapists working in public-sector hospitals had experience working internationally in ICUs (Table 2).

**TABLE 2:** Profile of physiotherapists working in public-sector physiotherapy departments which service intensive care units.

Physiotherapists characteristics	*n* = 429	Proportion (%)
**Age categories**		
20–30	**265**	**62**
31–40	116	26
41–50	24	6
51–60	20	5
61–65	4	1
**Level of training**		
Diploma	6	1.4
Bachelor	**408**	**95.1**
Master’s (not ICU-specific)	14	3.3
Master’s (ICU-specific)	1	0.2
PhD (ICU-specific and not)	0	0
**Distribution of physiotherapists per province**		
Eastern Cape	51	12
Free State	39	9
Gauteng	**107**	**25**
KwaZulu-Natal	**99**	**23**
Limpopo	30	7
Mpumalanga	17	4
Northern Cape	21	5
North West	13	3
Western Cape	52	12
**Post level**		
Assistant director	31	7
Production Level 3 chief	73	17
Production Level 2 senior	108	25
Production Level 1 junior	**133**	**31**
Community service physiotherapists	84	20
**International ICU work experience**		
Yes	34	8
No	66	92

Note: Bold font refers to the majority total for each characteristic.

ICU, intensive care units.

All physiotherapy departments completing the survey reported that they were active in providing opportunities for staff to become involved in postgraduate activities specifically focused on ICU care (Table 3). It was interesting to note that 7% (*n* = 3) of departments reported that staff were actively involved in research projects and/or journal clubs, while 39% (*n* = 18) of hospitals reported that their staff have completed the ICU refresher course provided by the South African Physiotherapy Society (Table 3).

**TABLE 3:** Cardiopulmonary physiotherapy development opportunities provided by physiotherapy departments.

Activities	Number of departments providing opportunities *n* = 46	
	%	*n*
Research	**7**	**3**
Workshops	35	17
Journal club CPD	**7**	**3**
Conference attendance	13	6
Refresher course (paediatrics)	33	16
Refresher course (adult)	**39**	**18**
SA Physiotherapy Society CPR	17	8

Note: Bold font refers to the majority total for each characteristic.

CPD, continuous professional development; CPR, cardiopulmonary rehabilitation; SA, South Africa.

## Discussion

This is the first article that we are aware of that provides an overall view of the organisation and structure of physiotherapy departments functioning within public-sector hospitals in South Africa responsible for servicing ICUs. The importance of the service provided by hospital-based physiotherapy departments is increasingly being recognised, and work has been undertaken to define quality criteria (Steenbruggen et al. 2020). Our data highlight concerns regarding the context within which a physiotherapy service is provided in public-sector hospitals in SA, which could also affect the physiotherapy service provided to critically ill patients. The concerns are related to (1) the workload faced by physiotherapists; (2) the profile of the physiotherapists and (3) the post levels available within the public-sector hospitals.

Data on staffing ratios for allied health professions, specifically physiotherapy, both internationally and locally, are scarce and lag behind the nursing and medical fields (Cartmill et al. 2012; Fisher et al. 2012; Garland & Gershengorn 2013; Mackay & Ellis 2002). The European Society of Intensive Care Medicine (ESICM) has recommended a dedicated physiotherapist per 12-bed ICU (Gosselink et al. 2008). However, our data indicate that the majority of physiotherapy departments are not in a position to dedicate a physiotherapist to each 12-bed ICU as, in addition to ICU care, each physiotherapist is also responsible for providing care to over 50 more patients nursed in hospital each day. This is based on the ratio of one physiotherapist to 69 beds, as per the results of our study. While one can argue that not all patients nursed in a hospital are in need of physiotherapy, most studies have linked the increased availability of physiotherapy with improved patient outcomes (Merino-Osorio et al. 2018; Mackay & Ellis 2002; Oldmeadow, McBurney & Robertson 2002). Hospital-based physiotherapists are widely recognised as important members of the multidisciplinary team, ensuring maximal functional ability of hospitalised patients (Bonvento et al. 2017; Maddocks et al. 2018; Steenbruggen et al. 2020; Van Aswegen et al. 2017; Vincent-Onabajo, Mustapha & Oyeyemi 2014). The early management of acutely ill patients has been described as the start of recovery, and early intervention has been linked to a positive recovery trajectory (De Morton et al. 2007; Mackay & Ellis 2002; Wang, Wu & Wang 2018). With the high bed-to-therapist ratio, the public-sector physiotherapists may struggle to provide early physiotherapy care to all acutely ill patients, and this may affect outcomes.

The complexity of the hospitalised patient population adds to workload concerns. Largely driven by the escalating costs of healthcare and the large demands placed on the availability of acute beds, patients are often discharged as soon as they are physiologically stable, thus forcing the physiotherapist to prepare the patient and the family to manage without professional assistance at home (Morris et al. 2021; Wheat 2013). The heavy burden of disease in SA and resultant multimorbidities add to the complexity of patient presentation. These complexities could have implications for the availability and quality of services physiotherapists can provide. Large bed-to-physiotherapy ratios mean that physiotherapists are faced daily with very difficult decisions regarding who receives care and what care the patients receive. Critically ill patients in the ICU present with complex conditions, including concomitant life-threatening pathologies (Delaney et al. 2008). They require multiple interventions and processes with coordinated care by healthcare professionals in the ICU multidisciplinary team who are experienced and trained and have decision support to improve outcomes of these patients (Delaney et al. 2008). The increased burden of care of ICU patients may affect the physiotherapy service delivery in these units because of increased care demands of the critical patients and the already high workload of physiotherapists, as noted in our findings.

The majority of the physiotherapists employed in the public-sector hospitals are young and at the start of their careers. Ntinga and Van Aswegen (2020), in a qualitative study conducted in one province of South Africa, reported that there was a difference in perception between junior and senior physiotherapy staff regarding teamwork in public and private hospitals. It was reported that the hierarchy within teams, the lack of familiarity with team members and the lack of knowledge of ICU equipment and ICU policies weighed heavily on the junior physiotherapy staff (Ntinga & Van Aswegen 2020). However, the senior physiotherapy staff valued avoiding errors by mitigating fatigue, reducing high staff turnover and opening communication lines between team members in the ICU (Ntinga & Van Aswegen 2020). The latter may affect physiotherapy service delivery, teamwork and quality of patient care in the ICU in the SA context. Employing experienced practitioners has been linked to improved patient outcomes and cost savings within a healthcare sector (Chipchase & Prentice 2006; Prentice & Chipchase 2006; Resnik & Jenson 2003). Researchers have previously determined that more experienced physiotherapists had a patient-centred approach to care, engaged in collaborative clinical reasoning and ensured the promotion of patient empowerment, when compared to novice physiotherapists. The age of the physiotherapists is related to the post levels available within the public-sector hospitals. It is unclear how the young physiotherapists employed in the public-sector hospitals in South Africa make the difficult decisions in caring for all the patients allocated to their care and how those decisions affect patient outcomes. Although Ntinga and Van Aswegen (2020) shed some light on the challenges faced by physiotherapists in this context, their study is focused on physiotherapists’ perceptions of collaborating with interprofessional team members in an ICU setting. More focus on the overall challenges, or facilitators, faced by junior and senior physiotherapists in the public sector in SA and its ICUs should be explored, since the majority (close to 80%) of the country relies on public-sector healthcare, as only 16% have medical aid contracts (Maphumulo & Bhengu 2019).

The final concern raised by our data is the lack of senior-level physiotherapy posts available within the public healthcare sector. Having more senior physiotherapists in this sector will lead to more senior staff in the ICU, which is a complex setting with patients with complex pathologies. Senior physiotherapists in this sector, including in ICUs, could positively affect patient outcomes through the training of junior staff to work safely and effectively in the ICU, including in after-hours service delivery (Ntinga & Van Aswegen 2020; Price & Reichert 2017). We argue that it is positive for newly qualified physiotherapists to be placed within an existing physiotherapy department to complete their community service. The environment lends itself to further training opportunities and mentoring of these young professionals (Price & Reichert 2017). However, this support comes at a price. In addition to the high bed-to-physiotherapist ratio, the complexity of the patients and the junior physiotherapists employed in this sector, the physiotherapy departments are also involved in the training of undergraduate students. Because of limited clinical placements available for the training of physiotherapists in South Africa, many of the hospitals will accept students from more than one university. Unlike clinical training in Australia and Canada (Alias et al. 2020; Jones et al. 2015; Newstead et al. 2017, 2019), clinical assessment and training is not standardised in SA, which could add to the burden of training students from different environments. Whether the investment into more senior public-sector posts could ensure stability for the training and mentoring of young professionals and improve patient outcomes needs to be investigated. Similar concerns have been raised by nursing colleagues working within the SA public healthcare sector, specifically as it relates to the management of patients in critical care (De Beer & Brysiewicz 2017; Scribante et al. 2004). They identified the challenging disease profile, the lack of resources because of the focus on primary healthcare, high patient-to-nurse ratios and lack of senior staff as barriers to providing optimal care in ICUs (Ntinga & Van Aswegen 2020; Scribante et al. 2004). It has been reported that ‘sufficient training and education to facilitate workplace transitions’ (Price & Reichert 2017:1) were expected by student and early-career nurses.

Student and early-career nurses also expected ‘continuing education opportunities throughout their careers for career laddering’ (Price & Reichert 2017:1), whereas the mid- to late-career nurses were reported to understand the importance of lifelong learning for ‘maintaining competency, providing quality patient care and enhancing career opportunities for the future’ (Price & Reichert 2017:1). In addition, they reported that the nurses felt that training and education provided career satisfaction (Price & Reichert 2017). Work environments that invested in continuing professional development opportunities to ensure continual growth in nursing practice and provide optimal quality care for patients were perceived by the nurses to be healthy work environments (Chen & Price 2020). In our study, there was minimal investment in research-related opportunities in the area of ICUs, with few postgraduate degrees specific to ICU research. Although a third of respondents claimed to provide the physiotherapists with the opportunity to attend the ICU Refresher Course, we did not explore the number of physiotherapists who actually attended this course. The latter may affect the provision of quality physiotherapy care for ICU patients, as well as the perception of physiotherapists of their work environment, in terms of support for continuing professional development and career development.

Our data must be interpreted with caution. We utilised a full population sampling methodology, and with a 70% completion rate, the data presented are a good reflection of the status in the sector. While four of the nine provinces reported a 100% response rate, one province had less than a 50% response rate. The survey was specific to the public sector and therefore gives a clear picture of this specific healthcare sector, as other studies have included both the private and public sectors, with a lack of differentiation between the two groups in their findings (Lottering & Van Aswegen 2016). The data are a snapshot of the sector at a specific time and could thus change over time. However, the trends regarding age; job description level; basic level of qualification; minimal ICU-specific CPD and postgraduate research; and physiotherapist-to-hospital bed ratio identified through the survey are reasons for concern.

## Conclusion

Our data provide insight into the organisational structure of physiotherapy departments and the physiotherapists working in the public-sector hospitals with ICU facilities. It is evident from the data that the therapists employed within this sector are young and early in their career development. The large number of ICUs functioning within these hospitals and the high bed-to-therapist ratio are concerning and highlight the high burden of care within this sector. Our data can now be used by researchers and universities to investigate the needs of public-sector physiotherapists and to explore the support needed from physiotherapy departments to facilitate student training, as well as by healthcare policymakers to guide service planning and delivery.
